# A case–cohort study for the disease natural history of adenoma–carcinoma and *de novo* carcinoma and surveillance of colon and rectum after polypectomy: implication for efficacy of colonoscopy

**DOI:** 10.1038/sj.bjc.6601007

**Published:** 2003-06-10

**Authors:** C-D Chen, M-F Yen, W-M Wang, J-M Wong, TH-H Chen

**Affiliations:** 1 Department of Family Medicine, Kaohsiung Medical University, Taiwan; 2 Institute of Preventive Medicine, College of Public Health, National Taiwan University, Taiwan; 3 Division of Gastroenterology, Department of Internal Medicine, Kaohsiung Medical University, Taiwan; 4 Department of Internal Medicine, National Taiwan University Hospital, Taiwan

**Keywords:** adenoma–carcinoma, *de novo* carcinoma, dwelling time, adenoma size, histological type, Markov model

## Abstract

The disease natural history of colorectal neoplasm regarding two opposing theories, adenoma–carcinoma sequence and *de novo* carcinoma theory, is controversial and rarely quantified. The aims of this study are therefore to estimate the dwelling times of adenoma–carcinoma sequence by adenoma size and histological type, taking *de novo* carcinoma into account. The efficacy of polypectomy was therefore estimated making allowance for two pathways. A case–cohort design, underpinning a cohort with 13 908 subjects (including 10 496 normal subjects, 2652 polyps, 760 colorectal cancers) who underwent the first examination of colonoscopy between 1979 and 1998, was devised to estimate parameters associated with two opposing theories by randomly selecting 305 normal subjects, 300 patients with polyps, and 116 colorectal cancers from the cohort. All the 2652 polyps were linked to national cancer registry to ascertain 25 invasive carcinomas after polypectomy. For the five-state model associated with adenoma size, dwelling times of small (0.6–1 cm) and large adenoma (>1 cm) are 7.75 and 5.27 years for the model without considering *de novo*, and 17.48 and 15.90 years for the model taking *de novo* carcinoma into account. Similar findings are observed for the model associated with histological type. The estimated proportions of *de novo* carcinoma are 31.87% from the model by adenoma size and 27.81% from the model by histological type. Compared to size less than 5 mm, patients with adenoma size between 6 and 10 mm and patients with adenoma size larger than 1 cm have 2.17-fold (0.67–10.74) and 4.25-fold (1.23–14.70), respectively, for the risk of malignant transformation. There are similar findings for the model by histological type. The estimates of overall efficacy of colonoscopy in reducing CRC is 73% for the model allowing for *de novo* carcinoma and 88% for the model without considering *de novo* carcinoma theory. The efficacy of diminutive adenoma and small adenoma increases with follow-up years, whereas the efficacy of large adenoma decreases with follow-up years. In conclusion, about 30% of cancers arising from *de novo* sequence are demonstrated. This finding, together with the adenoma–carcinoma sequence associated with adenoma size and histological type, is important for the estimation of dwelling times, the efficacy of colonoscopy, and the surveillance of polyp after polypectomy.

Although adenoma–carcinoma sequence was firmly established, the disease natural history of colorectal neoplasm has been challenged by the opposing theory of *de novo* carcinogenesis (Kudo *et al*, 2000). The adenoma–carcinoma sequence theory, in which cancer is thought to originate from the pre-existing adenoma, has been generally accepted since Muto *et al* study (1975). *De novo* carcinoma theory has been noted in several Japanese studies since 1987 (Kudo *et al*, 2000) and further confirmed in recent Western country studies (Bedenne *et al*, 1992; Stolte and Bethke, 1995). The tenor of *de novo* carcinoma theory is that many lesions of small colorectal early cancers of the superficial type with nonpolypoid form (Kuramoto and Oohara, 1989; Shimoda *et al*, 1989), flat elevation or depressed type are carcinomas without any adenomatous remnants (Kudo *et al*, 1995,^2000^). Genetic studies also support the view that there are at least two pathways that lead to the development of colonic cancer, including polyp-cancer sequence and *de novo* (Hirota *et al*, 1995; Tsujitani *et al*, 1996; Wada *et al*, 1996). While two opposing theories were confirmed by histopathological studies, the relative importance of the two theories associated with development of colorectal carcinoma is still fraught with uncertainty. *De novo* carcinoma accounting for development of colorectal carcinoma ranges from 20 to 90%. To assess whether colorectal cancers are derived from adenoma–carcinoma sequence or *de novo* carcinoma, most studies retrospectively examined adenomatous remnants on the basis of selected cancer cases rather than cases derived from a longitudinal healthy cohort. In addition, few studies quantified the progression rates pertaining to the development of colorectal carcinoma following two theories. While a longitudinal follow-up study requires long-term follow-up, it is therefore valuable to elucidate the disease natural history of colorectal neoplasm taking both pathways into account using an efficient cohort study design. To shed light on the disease natural history associated with the two theories, it is also important to assess the efficacy of endoscopy, partly because transition parameters of the disease natural history not only provide baseline estimates without being interrupted by treatment and partly because early detection of flat- or depressed-type cancer arising from *de novo* carcinoma is difficult with endoscopy.

In addition to carcinogenesis of colorectal cancer, dwelling times obtained from the disease natural history of progression from adenoma to cancer by adenoma size and histological type were not precisely estimated (Winawer *et al*, 1997). These findings are very helpful for the evaluation of surveillance of polyp after polypectomy, because tubular adenoma may have slow progression and too intensive follow-up for them after polypectomy seems redundant. On the contrary, patients with the large adenomatous polyp removed by polypectomy may need more intensive follow-up. Arguably, since flat- or depressed-type cancer is difficult to be detected with endoscopy, the proportion of *de novo* carcinoma may also affect the efficacy of polypectomy. The aims of this study are therefore

1. to estimate the progression rate or dwelling time from normal, through adenoma, and finally to invasive carcinoma, taking *de novo* carcinoma into account;
2. to estimate the progression rate or dwelling time from normal, through adenoma, and finally to cancer by adenoma size and histological type allowing for *de novo* carcinogenesis;
3. to estimate the annual malignant transformation rate after polypectomy by adenoma size and histological type;
4. to assess the efficacy of colonoscopy in reducing colorectal cancer, taking *de novo* carcinoma sequence into account, based on the comparison of (1) or (2) with (3).

## MATERIALS AND METHODS

### Study design and data sources

There are two major parts involved in this study, including the disease natural history, without being confounded by treatment and malignant transformation after polypectomy. Study design and data sources are individually described as follows.

#### The disease natural history of adenoma–carcinoma and *de novo* carcinoma

**Figure 1:**
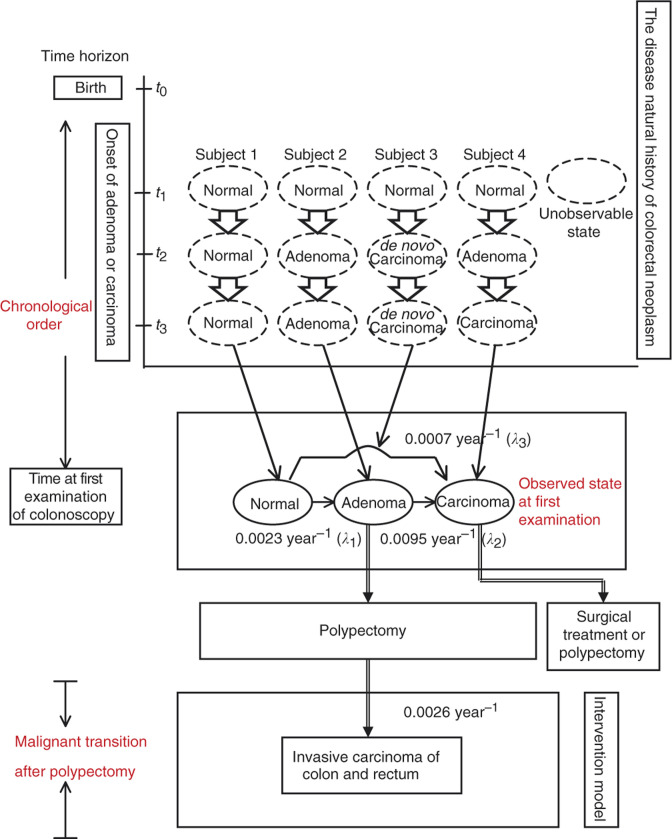
Clinical scenario of disease natural history model and intervention model for colorectal neoplasm.

As pointed out earlier, there are two possible pathways accounting for the occurrence of invasive carcinoma of colon and rectum. One of the pathways follows adenoma–carcinoma sequence, that is, normal→adenoma→carcinoma. The other is pertaining to the development of *de novo* cancers, like flat or depressed cancers without adenomatous remnants. To estimate the progression rates of adenoma–carcinoma sequence and direct transition to *de novo* cancer, one needs data on the first examination of colonoscopy because patients detected with adenoma or invasive carcinoma in the first examination were not confounded by treatment. This is illustrated in Figure 1

that shows four hypothetical cases associated with the disease natural history of adenoma–carcinoma and *de novo* carcinoma sequence before first colonoscopic examination. Chronological orders from birth (*t*_0_) to time window for the development of adenoma or carcinoma before first examination are depicted by three time points, including *t*_1_, *t*_2_, and *t*_3_. For simplicity, we assume that each individual was normal at *t*_1_. Subject 1 remained free of adenoma and invasive carcinoma before first examination. Subject 2 had onset of adenoma at *t*_2_ and dwelled in the state of adenoma without clinical symptoms until first examination. Subject 3 had depressed nonpolypoid early colon cancer arising from *de novo* at *t*_2_ and remained asymptomatic before first examination. Subject 4 had onset of adenoma at *t*_1_ and progressed to early colon cancer through adenoma–carcinoma sequence at *t*_3_, but remained asymptomatic before first examination. The middle panel in Figure 1 shows the observed detection modes of normal, adenoma, invasive carcinoma arising from *de novo*, and invasive carcinoma through adenoma–carcinoma sequence. The application of case–cohort design and the Markov model (see below) enables one to estimate the transition rates (average dwelling time, i.e. the inverse of transition rate) of the disease natural history of the two pathways. Taking *de novo* carcinoma sequence into account, the three-state model is further extended to a five-state model by dividing adenoma into three categories, diminutive (⩽5 mm), small (6–10 mm), and large (>10 mm) according to Read *et al* (1997) or by histological types (tubular (T), tubulovillous (T+V), and villous (V)).

**Figure 2:**
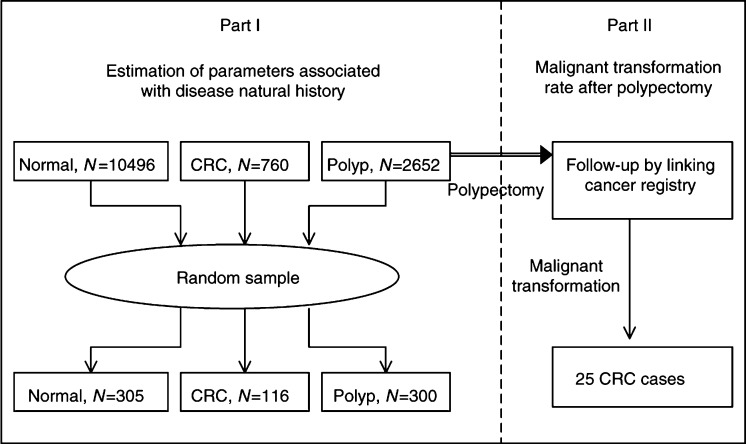
Study design and selection of samples for estimation of parameters associated with disease natural history and follow-up of polyps after polypectomy.

Data sources for estimating transition parameters were derived from a total of 13 908 subjects who underwent the first examination of colonoscopy between 1979 and 1998 in Kaushouing Medical center, the largest hospital in southern Taiwan. The reason of selecting one medical center is to ensure that the quality of skill did not vary greatly across colonoscopists. After receiving colonoscopy, this cohort includes three groups, 10 496 normal subjects, 2652 polyps, and 760 CRC cases (excluding HNPCC and FAP cases). Since the majority of subjects were examined between 1990 and 1998, the average follow-up years of this cohort excluding 760 cancers until the end of 1998 is 4.14 years, with 4.28 years for the normal group and 3.58 years for patients with the initial diagnosis as polyps. It should be noted that these 760 cancers might consist of cancers arising from adenoma–carcinoma sequence and *de novo* cancers. However, as some pathological reports did not ascertain whether invasive tumours contain any adenomatous remnants, we could not distinguish *de novo* cancers from cancers from adenoma–carcinoma sequence. In addition, since our pathological reports had not been computerised, instead of collecting pathological findings for all adenoma we used an efficient epidemiological design, a case–cohort design, to review and collect a set of random samples. Given 90% power and 5% statistical significant level, sample sizes using the ratio of approxi-mately 1 : 2 for normal or adenoma *vs* cancer were calculated. These include 305 normal subjects and 300 polyps that had not yet progressed to invasive carcinoma until the end of 1998 were randomly selected from normal and polyp cohorts, respectively. As regards CRC, a total of 150 cancers were randomly selected. How-ever, only 116 cases have complete information on pathological finding. The overall process is diagrammed in Part I of Figure 2

.

Demographic characteristics and endoscopy records for all subjects who underwent colonoscopy were reviewed and collected. Table 1

**Table 1:** Age, gender distribution, transition mode, and conditional transition probability by disease status of colorectal neoplasm from selected samples

**Disease type**	**Number of selected samples**	**Age (mean±s.d.)**	**Male/female subject**	**Transition mode**	**Conditional transition probabilities^a^**
Normal	305	49.57±15.15	0.93	Normal→Normal	*P*_1_^*^(*t*)
Polyp	300	57.14±13.60	1.34	Normal→Polyp	*P*_2_^*^(*t*)
Invasive carcinoma	116	60.56±13.27	1.52	Normal→Invasive carcinoma^b^	*P*_3_^*^(*t*)

a For the formula, see Appendix A;

b Including adenoma–carcinoma sequence and *de novo* carcinoma sequence.

shows the distribution of gender and age by disease status. The mean values of age are 49.57 (±15.15) for normal subjects, 57.14 (±13.60) for patients with polyps, and 60.56 (±13.27) for colorectal cancers. Sex ratios (male/female) are 0.93 for normal subjects, 1.34 for patients with polyps, and 1.52 for colorectal cancers. After selecting a subset of cohort, polyp size and histological type were abstracted from medical charts and pathological reports. Of 300 polyps, only 255 polyps had information on polyp size (Table 2

**Table 2:** The frequencies of polyps by size and histological type

**Clinical attributes**	**Frequency**	**Percentage (%)**
*Size groups*		
Diminutive (0.1–0.5 cm)	204	80.0
Small polyps (0.6–1.0 cm)	28	11.0
Large polyps (>1.0 cm)	23	9.0
Subtotal	255	
		
**Pathological type**		
*Adenoma*	119	64.3
Tubular	68	36.8
Tubulovillous	24	13.0
Villous	9	4.9
Adenoma (unclassified)	18	9.7
Nonadenomatous polyp	66	35.7
Subtotal	185	

). Polyps include adenomatous polyps and nonadenomatous polyps. Adenomatous polyps include three types, tubular (T), tubulovillous (T+V), and villous (V) (Read *et al*, 1997). Only 119 adenomatous polyps have information on histological types and advanced colorectal cancers. As we used a modelling method to estimate the proportions of *de novo* carcinoma and adenoma–carcinoma sequence, we included all colorectal invasive carcinoma including early and advanced cancers as end point. All colorectal cancers in our study were diagnosed according to the pathological findings in the light of Duke's stage classification. Early colorectal cancers are defined as Duke's stage A (limited to mucosa). Advanced cancer may include B (into muscularis propria), C (regional nodal metastases), and D (distant metastases).

#### Malignant transformation after polypectomy

The bottom panel of Figure 1 shows clinical scenario for the malignant transformation after polypectomy while adenomas were found. To ascertain malignant transformation, all patients with polyps after polypectomy in this cohort were linked to cancer registry data until the end of 1998 (Part II of Figure 2). A total of 25 CRC cases were ascertained with 35.71% diminutive adenoma, 28.57% small adenoma, and 35.71% large adenoma in terms of adenoma size, and 57.14% tubular adenoma, 28.57% tubulovillous adenoma, and 14.29% villous adenoma in terms of histological type. These cases may be derived either from adenoma that overlooked in colonoscopic examination, from recurrent adenoma depending on adenoma size and histological type or *de novo* cancers that were not diagnosed while colonoscopy was applied. These cases were used to assess the effect of adenoma size and histological type on the malignant transformation rate after polypectomy.

### Statistical method

To elucidate the disease natural history of colorectal neoplasm for both cancers arising from adenoma–carcinoma sequence and cancers arising *de novo* (i.e. without any adenomatous component), we proposed a three-state Markov model including the progressions of adenoma–carcinoma sequence and *de novo* carcinoma (see the middle panel of Figure 1).

Annual incidence rate of adenoma (*λ*_1_) is defined as the force of entering into adenoma per year. Annual transition rate from adenoma to cancer (*λ*_2_) is defined as the force of progression from adenoma to cancer per year. Annual incidence rate from normal to *de novo* carcinoma (*λ*_3_) is defined as the force of entering into *de novo* carcinoma per year. Since only adenomas were modelled, we assume that the disease is irreversible. The five-state Markov models by adenoma size, normal→diminutive→small→large→invasive carcinoma, and by histological types (normal→T→T+V→V→invasive carcinoma) plus the pathway of direct transition from normal to *de novo* carcinoma. There are two rationales for using Markov models. First, since multistate transitions rather than traditional two-state transition (normal→disease) are involved, the Markov models are one of the approaches for such a purpose. Second, the Markov model not only deals with disease progression from different pathways, but also accommodates unobservable transition like adenoma to carcinoma due to the interruption of treatment. The detailed algebra of three-state Markov models taking *de novo* carcinoma into account is presented in Appendix A. Due to the Markov property that assumes that the next transition is dependent on the current transition but independent of the previous transition, annual transition rate follows an exponential distribution. Consequently, the inverse of annual transition rate gives an average dwelling time staying in each state. The detailed methodology related to the application of Markov model to the disease natural history refers to Duffy *et al* (1995) and Chen *et al* (2000). Estimation of parameters and the proportion of *de novo* carcinoma are illustrated in Appendix A.

Estimation of parameters on malignant transformation after polypectomy is based on accelerated failure time (AFT) model with exponential distribution (Collett, 1994). To assess the efficacy of polypectomy, one can calculate one minus the ratio of 5- or 10-year probabilities of malignant transformation rate after polypectomy (*P*_T_) to the corresponding transition probabilities pertaining to the disease natural history (*P*_N_), see Appendix A (Treatment efficacy=1−*P*_T_/*P*_N_).

## RESULTS

### Descriptive results

Table 1 shows that adenoma and cancer are older than subjects free of colorectal neoplasm. Patients with cancer are slightly older than patients with adenoma. This suggests that the disease process from normal, adenoma, and finally to invasive carcinoma is progressive. Male subjects have more preponderance in adenoma and cancer than females subjects. Table 2 shows the frequencies of distribution by polyp size and histological type. Most polyps are of diminutive polyps (size smaller than 5 mm in diameter) and tubular adenoma. There is a significant relation between size and histological type (*χ*^2^=14.81; *P*=0.022) (data not shown). The larger polyp is more likely to have villous structure than the smaller one.

### Natural history of adenoma–carcinoma of CRC

Table 3

**Table 3:** Estimated results of natural history of adenoma–carcinoma of colon and rectum by three Markov models

**Transition mode**	**Estimate**	**95% CI**
*Three-state model*		
Normal→adenoma	3.1 × 10^−3^	2.6 × 10^−3^–3.6 × 10^−3^
Adenoma→invasive CRC	2.2 × 10^−2^	1.6 × 10^−2^–2.4 × 10^−2^
		
*Five-state model by adenoma size*		
Normal→diminutive	3.1 × 10^−3^	2.6 × 10^−3^–3.6 × 10^−3^
Diminutive→small adenoma	3.8 × 10^−2^	3.0 × 10^−2^–4.7 × 10^−2^
Small→large adenoma	1.3 × 10^−1^	7.8 × 10^−2^–1.8 × 10^−1^
Large adenoma→invasive CRC	1.9 × 10^−1^	9.5 × 10^−2^–2.8 × 10^−1^
		
*Five-state model by histological type*		
Normal→tubular adenoma	3.1 × 10^−3^	2.6 × 10^−3^–3.6 × 10^−3^
Tubular adenoma→tubulovillous adenoma	3.8 × 10^−2^	2.9 × 10^−2^–4.7 × 10^−2^
Tubulovillous adenoma→villous adenoma	1.1 × 10^−1^	6.5 × 10^−2^–1.5 × 10^−1^
Villous adenoma→invasive CRC	2.8 × 10^−1^	1.0 × 10^−1^–4.6 × 10^−1^

shows the estimated results of three- and five-state natural history models by adenoma size and histological types without considering *de novo* carcinoma. For the three-state model, annual incidence rate of adenoma is 3.1 × 10^−3^. Annual transition rate from adenoma to cancer is 2.2 × 10^−2^. For the five-state model depicted by adenoma size, annual transition rate from diminutive to small adenoma is 3.8 × 10^−2^. Annual transition rates from small adenoma to large adenoma and from large adenoma to cancers are 1.3 × 10^−1^ and 1.9 × 10^−1^, respectively. The inverse of both these figures gives 7.75 (1/0.1291) years and 5.27(1/0.1897) years of the corresponding dwelling times.

For the five-state model associated with histological type, annual transition rate from tubular to tubulovillous is 3.8 × 10^−2^. Annual transition rates from tubulovillous to villous and from villous to cancers are 1.1 × 10^−1^ and 2.8 × 10^−1^, respectively. Similarly, the estimates of dwelling time for tubulovillous type and villous type are 9.46 (1/0.1057) years and 3.60 (1/0.2779) years, respectively.

### Natural history of colorectal neoplasm taking *de novo* cancer into account

Table 4

**Table 4:** Estimated results of natural history of colorectal cancer, taking *de novo* into account, by three Markov models

**Transition mode**	**Estimate**	**95% CI**
*Three-state model*		
Normal→adenoma	2.3 × 10^−3^	1.3 × 10^−3^–3.4 × 10^−3^
Adenoma→invasive CRC	9.5 × 10^−3^	−5.5 × 10^−3^–9.5 × 10^−3^
Normal→CRC (*de novo*)	7.3 × 10^−4^	−2.4 × 10^−4^–1.7 × 10^−3^
		
*Five-state model by adenoma size*		
Normal→diminutive	2.1 × 10^−3^	1.4 × 10^−3^–2.8 × 10^−3^
Diminutive→small adenoma	2.1 × 10^−2^	8.6 × 10^−3^–3.3 × 10^−2^
Small→large adenoma	5.7 × 10^−2^	5.5 × 10^−3^–1.1 × 10^−1^
Large adenoma→invasive CRC	6.3 × 10^−2^	−2.9 × 10^−2^–1.5 × 10^−1^
Normal→CRC (*de novo*)	9.5 × 10^−4^	3.6 × 10^−4^–1.5 × 10^−3^
		
*Five-state model by histological type*		
Normal→tubular adenoma	2.2 × 10^−3^	1.4 × 10^−3^–3.0 × 10^−3^
Tubular adenoma→tubulovillous adenoma	2.3 × 10^−2^	8.9 × 10^−3^–3.7 × 10^−2^
Tubulovillous adenoma→villous adenoma	5.1 × 10^−2^	1.0 × 10^−3^–1.0 × 10^−1^
Villous adenoma→invasive CRC	1.1 × 10^−1^	−4.14 × 10^−2^–2.6 × 10^−1^
Normal→CRC (*de novo*)	8.6 × 10^−4^	1.9 × 10^−4^–1.5 × 10^−3^

shows the estimated results by taking *de novo* cancer into account. For the three-state model, annual incidence rate of adenoma is 2.3 × 10^−3^. Annual transition rate from adenoma to cancer is 9.5 × 10^−3^. Annual transition rate to *de novo* cancers is 7.3 × 10^−4^. Using expression (A.5) (see Appendix A) yields 23.78% of the proportion of *de novo* cancer. For the five-state model associated with adenoma size, after considering *de novo* cancer, annual incidence rate of adenoma is about 2.1 × 10^−3^. Annual transition rate from diminutive to small adenoma is 2.1 × 10^−2^. Annual transition rates from small to large adenoma and large adenoma to invasive carcinoma are reduced to 5.7 × 10^−2^ and 6.3 × 10^−2^. The inverse of both these figures gives 17.48 and 15.90 years of the corresponding dwelling time. Annual transition rate to *de novo* cancers is 9.5 × 10^−4^. This gives 31.87% of cancers arising from *de novo* sequence.

For the five-state model associated with histological type, annual transition rate from normal to tubular adenoma is 2.2 × 10^−3^. Taking *de novo* cancer into account, annual transition rate from tubular to tubulovillous adenoma is 2.3 × 10^−2^. The annual transition rates from tubulovillous to villous adenoma and villous adenoma to invasive carcinoma are 5.1 × 10^−2^ and 1.1 × 10^−1^. The inverse of both these figures gives 19.80 and 8.98 years of the corresponding dwelling time. Annual transition rate to *de novo* cancers is 8.6 × 10^-4^. This gives 27.81% of cancers arising from *de novo* sequence.

### Transition rate after polypectomy of CRC

Of 2652 polyps, 25 cancers were ascertained with 3.58 average years of follow-up. This gives 2.6 × 10^−3^ (25/(9494=2652 × 3.58)) of annual malignant transformation rate after polypectomy using accelerated failure time model.

Compared to size less than 5 mm, patients with adenoma size between 6 and 10 mm and larger than 1 cm have 2.17-fold (0.58–8.08) and 4.25-fold (1.23–14.70), respectively, for the risk of malignant transformation (Table 5

**Table 5:** Estimates of parameters on malignant transformation by adenoma size and histological type after polypectomy, using accelerated failure time (AFT) model

**Variable**	**Hazard ratio**	**95% CI for hazard ratio**
*Size*		
0–0.5 cm	1.00	–
0.6–1 cm	2.17	0.58–8.08
>1 cm	4.25	1.23–14.70
		
*Histological type*		
Tubular adenoma	1.00	–
Tubulovillous adenoma	1.51	0.45–5.01
Villous adenoma	2.53	0.54–11.91

). The estimates of relative risk for malignant transformation for tubulovillous and villous types are 1.51 (95% CI: 0.45–5.01) and 2.53 (95% CI: 0.54–11.91) compared to tubular adenoma.

### Evaluation of efficacy for polypectomy

The ratio of annual malignant transformation rate after polypectomy to the progression from adenoma to cancer in the light of disease natural history in Table 3 without taking *de novo* cancers into account gives 88% ((1−0.0026/0.022) × 100%) as the efficacy of polypectomy.

**Figure 3:**
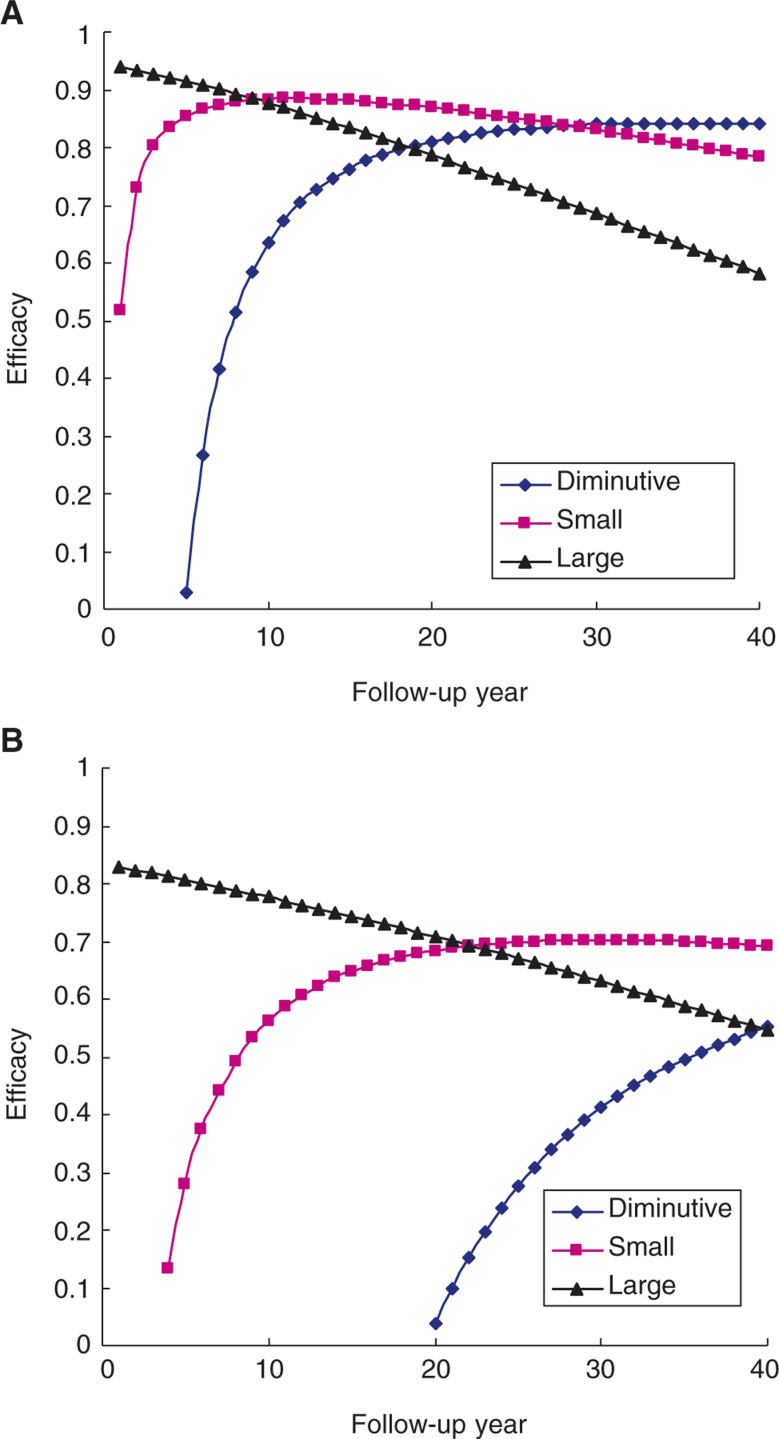
(**A**) Efficacy of reducing CRC with follow-up years by adenoma size; (**B**) Efficacy of reducing CRC with follow-up years by adenoma size, taking *de novo* into account.

Taking adenoma size into account, Figure 3A

shows the efficacy of diminutive and small adenoma increases with follow-up years and reaches full benefit around 10 and 20 years of follow-up, whereas the efficacy of large adenoma decreases with follow-up years. The efficacy of diminutive adenoma increases from 2.76% at 5 years to 63.54% at 10 years. The efficacy of large adenoma is reduced from 91.47% at 5 years to 78.62% at 20 years. Similar results are also found for the efficacy pertinent to histological type. The efficacy for diminutive adenoma increases from 18.64% at 7 years to 48.03% at 10 years. The efficacy for villous adenoma is reduced from 93.68% at 5-year to 80.94% at 20-year follow-up. Taking *de novo* cancer into account, the efficacy of polypectomy is reduced. The overall efficacy of polypectomy is reduced to 72.63% ((1−0.0026/0.0095) × 100%) compared to 88%, without considering *de novo* cancer. The absolute efficacy in Figure 3B is smaller than that in Figure 3A. However, the pattern of the efficacy of polypectomy by adenoma size in Figure 3B is similar to that in Figure 3A. The estimates of efficacy of polypectomy for large adenoma are 80.64% after 5-year and 70.80% after 20-year follow-up, taking *de novo* sequence into account. The estimates of efficacy of polypectomy for villous adenoma are 88.31% after 5 years and 78.71% after 20 years, taking *de novo* sequence into account.

## DISCUSSION

Colorectal neoplasm associated with the disease natural history, the surveillance after polypectomy, and the efficacy of colonoscopic examination were elucidated in this study. Using Markov models, the first part quantified the disease natural history of colorectal neoplasm from two pathways, the established adenoma–carcinoma sequence and *de novo* cancers that have been observed to arise in flat mucosa without adenomatous remnants. Adenoma size and histological type were also taken into account while the disease natural history of adenoma–carcinoma sequence was modelled.

In addition to modelling the disease natural history of CRC based on data from first colonoscopic examination, the second part is related to the estimation of the overall annual malignant transformation rate and the corresponding rates by adenoma size and histological type after polypectomy.

The third part is to estimate the efficacy of colonoscopy in reducing malignant transformation on the basis of parameters from the first part of the disease natural history and the second part of malignant transformation after polypectomy. Results from the three parts can yield several significant implications for clinical practice, including dwelling time of adenoma by size and histological type, the proportion of cancers arising from *de novo* cancers, the surveillance of adenoma after polypectomy, and the efficacy of colonoscopy. These aspects associated with the disease natural history model are discussed as follows.

### Dwelling time of adenoma

The disease natural history model elucidated in this study can throw light on the dwelling time of staying in precancerous lesion by adenoma size and histological type. Without considering *de novo* cancer sequence, the dwelling times are 26 years for diminutive adenoma, 8 years for small adenoma, and 5 years for large adenoma, respectively. The corresponding figures are 26 years for tubular adenoma, 9 years for tubulovillous adenoma, and 4 years for villous adenoma. Taking *de novo* cancer into account, the dwelling times of adenoma are expected to be longer, with 48 years for diminutive adenoma, 17 years for small adenoma, and 16 years for large adenoma, respectively. The corresponding figures are 44 years for tubular adenoma, 20 years for tubulovillous adenoma, and 9 years for villous adenoma. The above estimates of dwelling time of adenoma–carcinoma suggest a long window period for early detection of adenoma using colonoscopy. However, while *de novo* cancers are taken into account, the dwelling times are longer. This suggests that detecting *de novo* cancer by colonoscopy, possibly, with the spraying method cannot be overemphasised. However, the efficacy of colonoscopy in reducing cancers is highly dependent on the proportion of *de novo* cancers (see below).

### Adenoma–carcinoma or *de novo* cancer

Although two possible pathways, adenoma–carcinoma or *de novo* cancer sequence, have been proposed, the relative importance of both pathways to cancer has been rarely addressed. After taking adenoma size or histological type into account, the present study used five-state Markov models to identify around 30% cancers of colon and rectum arising from *de novo* carcinoma. Using transition parameters from the Markov model enables one to predict the risk of getting *de novo* cancer. One-, 3-, 5-, 10-, and 20-year probabilities from normal to *de novo* cancer were 0.15, 0.45, 1.5, and 2.9%, respectively, which are higher than the corresponding figures 2.2 × 10^−5^, 2.0 × 10^−4^, 5.4 × 10^−4^, 2.1 × 10^−3^, and 7.8 × 10^−3^ for cancer arising from adenoma–carcinoma sequence.

### Surveillance of adenoma after polypectomy

The overall efficacy in reducing CRC with colonoscopy is 88%. Taking *de novo* carcinoma into account, the efficacy is reduced to 73%. However, the efficacy of reducing CRC with polypectomy is also contingent on size and histological type. The hazard ratios in Table 5 show that large adenomatous polyps or villous adenoma have a higher malignant transformation than diminutive, small, or tubular adenoma. Taking these findings into account, there is a decreasing linear relationship of the efficacy of polypectomy by follow-up years for large adenomatous polyps but an increasing trend with nonlinear relation for diminutive or small adenomatous polyps (Figure 3A). Large adenomatous polyps have short dwelling time leading to high potential of malignant transformation. Consequently, the full efficacy is observed immediately after polypectomy and decreases with follow-up years. In contrast, dwelling times for diminutive and small adenoma are so long as to be unlikely to yield benefit in short-term follow-up in comparison with those without polypectomy. Full benefits are therefore reached after 10 years of follow-up for small adenoma and 20 years of follow-up for diminutive adenoma. Similar findings are also observed for histological type. The similar pattern, albeit the efficacy is reduced, is observed while *de novo* carcinoma is considered. The above findings suggest that the efficacy of polypectomy is not only dependent on the proportion of cancers arising from *de novo* carcinoma, but also varies according to adenoma size and histological type.

### Comparison with previous findings

Earlier studies on *de novo* carcinoma yielded varied results with proportions of *de novo* cancers ranging from 20 to 90%. The discrepancies across studies may be due to the difference between the series in methods and in selection cases. Our finding of 30% colorectal carcinoma arising from *de novo* carcinoma is slightly lower than Bedenne *et al*'s (1992) result that was derived from a nonselected population-based series of 1630 resected colorectal cancer and much lower than the finding of 80–90% colorectal carcinoma arising from *de novo* carcinoma sequence in Japanese studies. Since our study was not based on evaluation of adenomatous remnants of colorectal carcinoma, the misclassification of *de novo* cancers such as flat-type into diminutive or small adenoma due to being devoid of using the dye-spraying technique in the present study provides an alternative explanation for lower proportion of *de novo* carcinoma as compared with the Japanese studies. This should be further corroborated in the ongoing study. Nonetheless, our finding still suggests at least 30% colorectal cancers arising from *de novo* carcinoma sequence.

If the disease natural history follows adenoma–carcinoma theory, annual transition rate from adenoma to carcinoma is 2.20%, which is close to previous findings, including 1.99% in Muto *et al* study (1975), 2.15% in NPS study (O'Brien *et al*, 1990), and 2.15% in Stryker *et al* study (1987), respectively.

Annual malignant transformation rates by size and histological type are also in agreement with previous findings that the malignancy rate is higher in large adenomas and adenoma with villous structure than in adenoma without exhibiting these features (Muto *et al*, 1975; Wolff and Shinya, 1978). However, as discussed in the previous part, the estimates of dwelling time will be reduced if *de novo* carcinoma theory is considered. This suggests that quantifying the disease natural history should take *de novo* carcinoma into account.

### Methodological consideration

In contrast to previous studies, the elucidation of the relative importance of adenoma–carcinoma sequence and *de novo* cancer was based on Markov models in our study (see Figure 1). The optimal estimates associated with both pathways were therefore obtained. The earlier studies on clarifying adenoma–carcinoma sequence or *de novo* cancer were, retrospectively, to collect cancers or flat cancer of colon and rectum and to ascertain *de novo* cancers or adenoma–carcinoma sequence by examining whether there were adenomatous remnants. However, the proportion of *de novo* carcinoma is, in fact, dependent on what sort of cancer or how large samples of cancers are collected. If flat cancers are more likely to be included, the larger proportion of *de novo* cancer will be obtained. This can account for why 80% flat cancers derived from *de novo* cancers were demonstrated in Shimoda *et al* (1989) and only 22% in Stolte and Bethke study (1995). As cancers in the present study were derived from a cohort receiving colonoscopy, they were composed of cancers from two pathways. Consequently, the estimate of 30% would have represented the proportion of subjects as the same cohort in the present study with the potential of the development of *de novo* cancer if they had chance of developing into cancer.

There are two limitations in this study. First, to uncover invasive carcinoma by linking all polyps with cancer registry may not only consist of missed polyps but also include new polyps, recurrent polyps, and possible *de novo* carcinoma that coexist with polyp and those undetected in colonoscopy examination. The efficacy of polypectomy in the current study may be related not only to lesions overlooked in colonoscopy examination but also recurrent polyps, new polyps, and *de novo* cancer. However, although our models can take *de novo* into account, they still have limitations in separating recurrent polyps or new polyps from missed polyps.

Second, we used information of age of diagnosis at first examination and different modes (normal, adenoma, and cancer), together with the technique of Markov model to yield the estimates of the disease natural history model including adenoma–carcinoma and *de novo* carcinoma. However, the Markov property needs to assume constant transition rates. This assumption, of course, may not be valid for cancer incidence but may be reasonable for adenoma for subjects aged over 50 years because they had already had adenoma before first examination. As the majority of subjects receiving colonoscopy in this cohort were older than 50 years, we believe the assumption may not be unreasonable. This can be supported by a good fit between the observed and the expected for the five-state Markov model-associated adenoma size (*χ*^2^=0.15, *P*=0.70).

In conclusion, the disease natural history of adenoma–carcinoma by adenoma size and histological type and *de novo* carcinoma sequence was quantified using Markov models. About 30% of cancers were estimated to arise from *de novo* sequence. Considerations of *de novo* carcinoma and adenoma–carcinoma sequence associated with adenoma size and histological type play an important role in the estimation of dwelling times, the efficacy of colonoscopy, and the surveillance of polyp after polytectomy.

## Appendix A. Derivation of transition matrix, transition probabilities, and the likelihood function of three-state Markov model for the disease natural history of colorectal neoplasm

### Transition Matrix

The three-state model for the disease natural history of colorectal neoplasm taking *de novo* carcinoma into account (Figure 1) is

The definition of *λ*_1_, *λ*_2_, and *λ*_3_ refers to the methodological section. The transition parameters of *λ*_1_, *λ*_2_, and *λ*_3_ can be written in a transition matrix form (Q):

### Derivation of transition probabilities

Following Duffy *et al* (1995) and Chen *et al* (2000), transition probabilities for the transition from one state to another are denoted as follows:

*P_ij_*(*t*) represents the risk of transition from state *i* to state *j*. For example, the risk of transition from normal to invasive CRC during time *t* is denoted by *P*_13_(*t*).

### Likelihood and estimation of parameter

As our study is based on a case–cohort design, the conditional probability for polyp (state 2) given whether the sample was selected (*S*=1) is required and denoted as follows:

*π*_1_ (=305/10496), *π*_2_ (=300/2652), and *π*_3_ (=116/760) are random sample fractions for normal, polyp, and invasive carcinoma, respectively.

Similar formulae are derived for normal (*P*_1_^*^(*t*)) and invasive carcinoma (*P*_3_^*^(*t*)). Given these probabilities, one can develop the likelihood function based on the frequency in Table 1. Let *n*_1_ (=305), *n*_2_ (=300), and *n*_3_ (=116) denote the number of normal, polyp, and invasive CRC, respectively. The likelihood function, given this information, is

where *m_i_* represents age at first examination of the *i*th individual and *δ_ij_* is an indicator for the *i*th individual in state *j* (*j*=1, 2, 3).

SAS/IML procedure was used to estimate maximum likelihood estimates (MLEs) and standard error of estimates in Tables 3 and 4.

### Calculation of *de novo* carcinoma (*P*)

Using transition parameters of *λ*_1_ and *λ*_3_, it is easy to show that the proportion of *de novo* carcinoma (*P*) is calculated by
